# Incorporating social opinion in the evolution of an epidemic spread

**DOI:** 10.1038/s41598-021-81149-z

**Published:** 2021-01-19

**Authors:** Alejandro Carballosa, Mariamo Mussa-Juane, Alberto P. Muñuzuri

**Affiliations:** Group of Nonlinear Physics, Institute CRETUS, Faculty of Physics, University of Santiago de Compostela, 15782 Santiago de Compostela, Spain

**Keywords:** Computer modelling, Differential equations, Dynamical systems, Epidemiology, Biological physics, Statistical physics, thermodynamics and nonlinear dynamics

## Abstract

The evolution of the COVID19 pandemic worldwide has shown that the most common and effective strategy to control it used worldwide involve imposing mobility constrains to the population. A determinant factor in the success of such policies is the cooperation of the population involved but this is something, at least, difficult to measure. In this manuscript, we propose a method to incorporate in epidemic models empirical data accounting for the society predisposition to cooperate with the mobility restriction policies.

## Introduction

Both the amount of interactions that an infected individual carries out while being sick and the reachability that this individual has within its network of human mobility have a key role on the propagation of highly contagious diseases. If we picture the population of a given city as a giant network of daily interactions, we would surely find highly clustered regions of interconnected nodes representing families, coworkers and circles of friends, but also several nodes that interconnect these different clustered regions acting as bridges within the network, representing simple random encounters around the city or perhaps people working at customer-oriented jobs. It has been shown that breaking down the connectivity of these networks of interactions by means of imposing social distancing and isolation measures represent one of the most effective ways to control the virulent spread of a contact-transmissible infectious disease such as the influenza virus or the current COVID-19 coronavirus^1–5^. For these policies to succeed however, it is needed that the majority of the population adheres willingly to them since frequently these contention measures are not mandatory and significant parts of the population exploit some of the policies gaps or even ignore them completely. In diseases with a high basic reproduction number, i.e., the expected number of new cases directly generated by one infected case, such is the case of COVID-19, these individuals represent an important risk to control the epidemic as they actually conform the main core of exposed individuals during quarantining policies. In case of getting infected, they can easily spread the disease to their nearest connections in their limited but ongoing everyday interactions, reducing the effectiveness of the social distancing constrains and helping on the propagation of the virus. Measures of containment and estimating the degree of adhesion to these policies are especially important for diseases where there can be individuals that propagate the virus to a higher number of individuals than the average infected. These are the so-called super-spreaders^6,7^ and are present in SARS-like diseases such as the COVID-19. Recently, a class of super-spreaders was successfully incorporated in mathematical models^8^.

Regarding the usual epidemiological models based on compartments of populations, a viable option is to introduce a new compartment to account for confined population^9^. Again, this approach would depend on the adherence of the population to the confinement policies, and taking into account the rogue individuals that bypass the confinement measures, it is important to accurately characterize the infection curves and the prediction of short-term new cases of the disease, since they can be responsible of a dramatic spread. The importance of individual opinions in social networks has been studied before^10^ and its role in epidemic propagation can be inferred from there.

Here, we propose a method that quantitatively measures the state of the public opinion and the degree of adhesion to an external given policy. Then, we incorporate it into a basic epidemic model to illustrate the effect of changes in the social network structure in the evolution of the epidemic. The process is as follows. We reconstruct a network describing the social situation of the Spanish society at a given time based on data from social media. This network is like a radiography of the social interactions of the population considered. Then, a simple opinion model is incorporated to such a network that allows us to extract a probability distribution of how likely the society is to follow new opinions (or political directions) introduced in the net. This probability distribution is later included in a simple epidemic model computed along with different complex mobility networks where the virus is allowed to spread. The framework of mobility networks allows the explicit simulation of entire populations down to the scale of single individuals, modelling the social structure of human interactions, mobility and contact patterns. This approach attempts to reproduce the face-to-face interactions that occur in the everyday of a population, and is able to easily accommodate real demographic information and actual individual interactions extracted from social networks such as Enron or Facebook^11^. These features make them a promising tool to study epidemiological models (see^12^ for a review), especially if we are interested in controlling the disease by means of altering the interaction patterns of individuals. At this point, we must highlight the difference between the two networks considered: one is collected from real data from social media and it is used to feel the mood of the collective society, while the other is completely in-silico and proposed as a first approximation to the physical mobility of a population.

In the literature, several methods to hinder an epidemic spread on complex networks have been developed and analyzed. To cite some, in^1^, they simulate a network of individuals in which connections and face-to-face interactions are dynamically cut down in the moment an individual gets sick. On the other hand, in^11,13,14^, the nodes with the highest degree of interactions are targeted with vaccination policies before the outbreak, in order to halt the future spreading of the virus making it more difficult for it to percolate through the network. These kind of strategies work by altering the topology of the network, blocking either the edges or the nodes, but alternatives that maintain the structure of the network intact are also feasible, such as introducing another diffusive element that competes with the already existing one. In^15^, they compare and analyze the effectiveness of both these methods in restraining diffusion, in the context of information propagation with a SIR-like model. In the present work, we explore a somehow similar approach of altering the topology of the mobility network as in^1^, by introducing the opinion distribution extracted from the social media as a modulator of the connectivity pattern of the network. However, instead of breaking down the connections of a node dynamically with the outbreak of the epidemic, we incorporate here social distancing as a prevention policy, in such way that each individual will modify its usual connections according to its predisposition to follow external directives.

The study case considered to exemplify our results considers the situation in Spain. This country was hard-hit by the pandemic with a high death-toll and the government reacted imposing a severe control of the population mobility. The policy worked and the epidemic was controlled, nevertheless it has been difficult to estimate the level of adherence to those policies and the repercussions in the sickness evolution curve. This effect can also be determinant during ulterior developments of the pandemic.

The manuscript is organized as follows. In “Methods” we describe the construction of the social network from scratch using free data from Twitter, the opinion model is also introduced here and described its coupling to the epidemiological model. "Results" contains the main findings and computations of the presented models, and "Discussion" a summary and a brief discussion of the results, with conclusions and future perspectives.

## Methods

### Social network construction

In order to generate a social network we use data from Twitter, a microblogging website that allows users to post their ideas and opinions in form of short messages or “tweets”. If a user’s account is public, the rest of the users are able to see his or her tweets and interact with them in different forms. In addition, if one user finds interesting the content of another user, the website allows them to follow each other so one receives the content published by the other every time they enter to the website. This kind of interactions converts Twitter into a massive web of users interacting with each other in several different topics every day, with hidden layers of structure given by the following processes, which are not directly shown in the website. Using the tool NodeXL^16^ and a given word or phrase of interest, we are able to collect a list of several users that have tweeted any message containing the typed word and the list of the users that interacted anyhow with those tweets. From this, the software builds a network with the users on its nodes and the interactions of any kind between users as its links. Furthermore, the tool can unveil if different users in the established network of interactions are following each other or if they have followers in common, developing local structures and communities among nodes. Using different words of interest, such as “#QuedateEnCasa” (#StayAtHome), #DiaMundialDeLaSalud (#WorldHealthDay) or “#EsteVirusLoParamosUnidos” (#TogetherWeFightThisVirus), we downloaded several networks and overlapped them to build a more complete network of connections. We tried to choose neutral topics with potentiality to engage many people independently of political commitment, age, or other distinctions. The examples above show that the topics are quite generic lacking political significance being, thus, good choices to involve a large representative number of users. Each one of the downloaded networks has approximately 2000 nodes^17^, which is one of the limitations of the software (it does not allow to download more than 2000 nodes per network). In any case, downloading as many of such subnets as possible gives us a more realistic map of the current situation of the Spanish Twitter network and, we believe, a realistic approximation to the social interactions nationwide.

We intended to download diverse networks politically inoffensive. ‘Junction’ accounts will be needed to make sure that all sub-networks overlap. Junction accounts are these accounts that are part of several subnets and warrant the connection between them. If these junction accounts did not exist, isolated local small networks may appear. A complete list of the words of interest used to download and overlap the networks is presented in the supplementary information. Note that the procedure here explained is a sort of snapshots of the complete Spanish social network that once accumulated in a single network provide us with a realistic picture of the social interactions. In the SI the effect of adding subnets to the total network is investigated and we noticed that the exponent of the network converges to a typical value after 7 to 10 subnetworks are considered.

Twitter, as a social network, changes in time^18–20^ and it is strongly affected by the current socio-political situation, so important variations in its configuration are expected with time. Specifically, when a major crisis, such as the current one, is ongoing. Taking this into consideration, we analyze two social networks corresponding to different moments in time. One represents the social situation in October 2019 (with $N=17665$ accounts) which describes a pre-epidemic social situation and another in April 2020 (with $N=24337$ accounts) which describes the mandatory-confinement period of time. The networks obtained are directed and the links mark which nodes are following or interacting with. So, a node with high connectivity means it is following the opinions of many other nodes or interacting heavily with the rest of the users.

**Figure 1:**
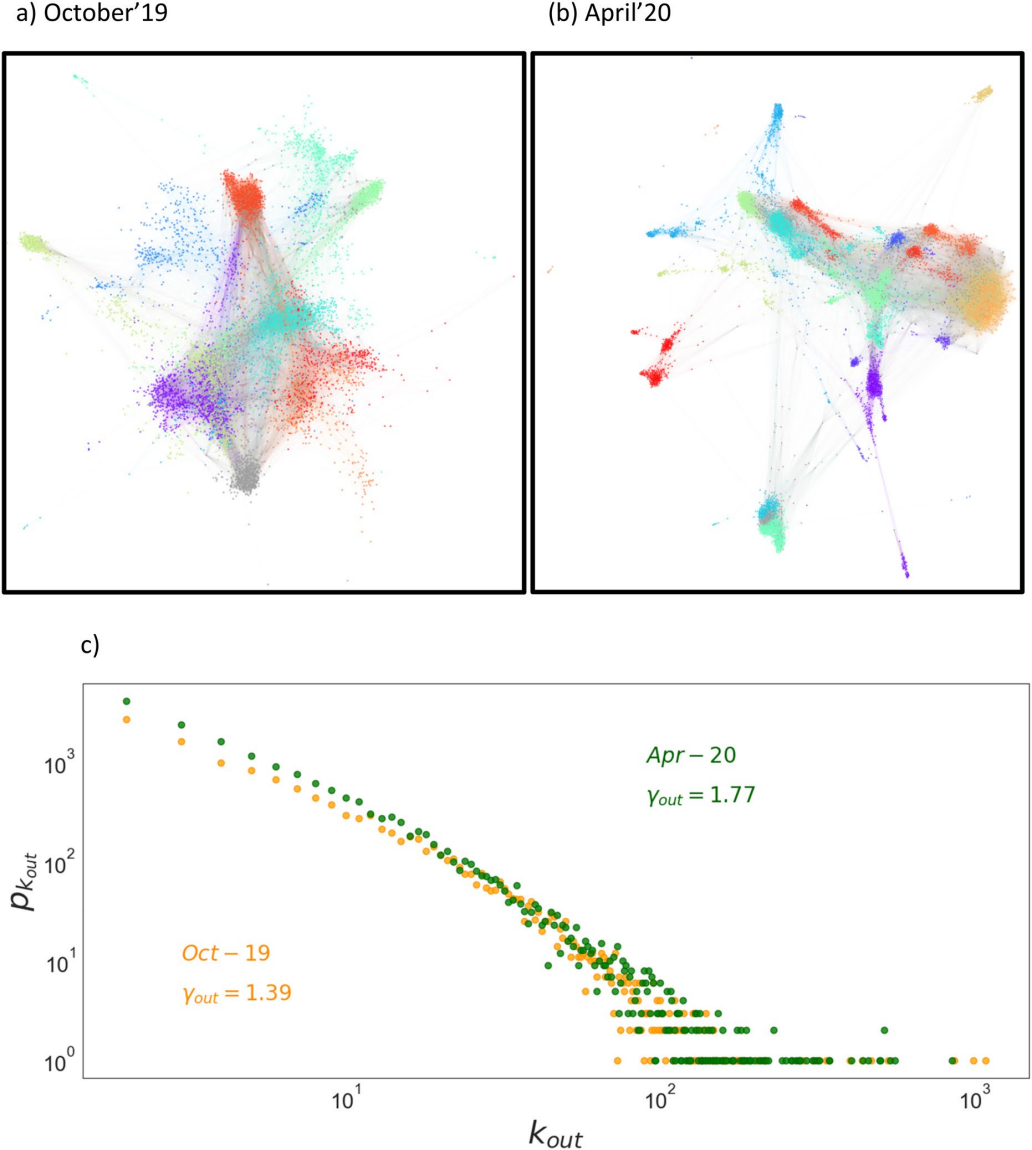
**(a)** October 2019 Twitter network. **(b)** April 2020 Twitter network. Each color marks those nodes corresponding with each word-of-interest subnet. Accounts in grey are the junction accounts. Links are colored with the origin node account. **(c)** Log–log plot of the connectivity distribution for both networks. We generate the graphs in **(a,b)** using the algorithm Force Atlas 2 from Gephi^22^. Force Atlas 2 is a forced-directed algorithm that stimulates the physical system to get the network organized through the space relying on a balance of forces; nodes repulse each other as charged particles while links attract their nodes obeying a Hooke’s law. So, nodes that are more distant exchange less information.

The two social networks obtained with this protocol are illustrated in Fig. 1. A first observation of their topologies demonstrate that they fit a scale free network with a power law connectivity distribution and exponents $\gamma =1.39$ for October’19 and $\gamma =1.77$ for April’20 network^21^. The significantly different exponents demonstrate the different internal dynamics of both networks. This difference will become more evident once an opinion model is considered in each network. The October 2019 network corresponds with a polarized political situation in the Spanish life with a second call for elections in a short time. While the April 2020 network corresponds with the hardest part of the pandemic wave hitting Spain. At that time, the population was shocked and very susceptible to follow orders by the government. This important difference is reflected in the different values of the network exponents.

A final remark should be made regarding the content of the downloaded network. We have discarded all the personal information related to the users and the content of their twits and retained only the topology of the subnetworks. Our aim is pointed towards finding the print that the social interaction pattern between individuals leaves on the topology of the network, and how this particular print is able to affect the dynamics of a given process, such as the opinion formation. However, note that the use of posts’ content could be used for similar purposes through means of sentiment analysis, although that is beyond the scope of the present work. For such an empirical analysis we refer the reader to^23^, where a larger number of Twitter posts is analyzed and the behavioral patterns of several users is studied.

### Opinion model

We consider a simple opinion model based on the logistic equation^24^ but that has proved to be of use in other contexts^25,26^. It is a two variable dynamical model whose nonlinearities are given by,

$$\frac{du}{dt}=f\left(u,v\right)=Au \left(1-\frac{u}{B}\right)+g\,u\,v$$

1 $$\frac{dv}{dt}=g\left(u,v\right)=Cv \left(1-\frac{v}{D}\right)-g\,u\,v$$

where $u$ and $v$ account for the two different opinions. $A, B, C, D$ and $g$ are model parameters whose meaning will be clear bellow. As $u+v$ remains constant, we can use the normalization equation $u+v=1$, and, thus, the system reduces to a single equation:

2 $$\frac{du}{dt}=f\left(u\right)=u\left[A\left(1-\frac{u}{B}\right)+g\left(1-u\right)\right]$$

$A$ is a time rate that modifies the rhythm of evolution of the variable $u$, $g$ is a coupling constant and $B$ controls the stationary value of $u$. Note that the parameters $C$ and $D$ from Eq. (1) have no role in the model in Eq. (2). This system has two fixed points (${u}_{0}=0$ and $u_{0}  = \frac{{A + g}}{{A{\text{/}}B + g}}$ being the latest stable and ${u}_{0}=0$ unstable.

We now consider that each node belongs to a network and the connections between nodes follow the distribution measured in the previous section. The dynamic equation for each one of the nodes ($i$) becomes^27^,

3 $$\frac{d{u}_{i}}{dt}=\dot{{u}_{i}}=f\left({u}_{i}\right)+d\frac{1}{{k}_{i}}\sum_{j=1}^{N}{L}_{ij}{u}_{j}$$

${u}_{i}$ describes the opinion state of node $i$. Each of the nodes $i$ obey the internal dynamic law given by $f\left({u}_{i}\right)$ (see Eq. (2)) while being coupled with the rest of the nodes with a strength $d/{k}_{i}$ where $d$ is a diffusive constant and ${k}_{i}$ is the connectivity degree for node $i$ (number of nodes each node is interacting with, also named outdegree). Note that this is a directed non-symmetrical network where ${k}_{i}$ means that node $i$ is following the Tweets from ${k}_{i}$ nodes. ${L}_{ij}$ is the Laplacian matrix, the operator for the diffusion in the discrete space, $i,j=1, \dots ,N$. We can obtain the Laplacian matrix from the connections established within the network as ${L}_{ij}={A}_{ij}-{\delta }_{ij}{k}_{i}$, being ${A}_{ij}$ the adjacency matrix

$${A}_{i,j}=\left\{\begin{array}{ll}1& \quad \text{if}\, i,\, j\,\text{are connected }(\text{user }i\text{ follows user }j)\\ 0& \quad \text{if}\,  i,\, j\, \text{are not connected }(\text{user }i\text{ does not follow user }j)\end{array}\right.$$

Notice that the mathematical definition in some references of the Laplacian matrix has the opposite sign. We use the above definition given by^27^ in parallelism with Fick’s law and in order to keep a positive sign in our diffusive system.

Now, we proceed as follows. We consider that all the accounts (nodes in our network) are in their stable fixed point ${u}_{0}=\frac{A+g}{g+\frac{A}{B}}$ , from Eq. (1), with a $10\%$ of random noise. Then a subset of accounts $r$ is forced to acquire a different opinion, ${u}_{i}=1$ with a $10\%$ of random noise, $\forall i / i = 1, ..rN$ and we let the system to evolve following the dynamical Eq. (3). In this case, accounts are sorted by the number of Followers that it is easily measured. Therefore, some of the nodes shift their values to values closer to 1 that, in the context of this simplified opinion model, means that those nodes shifted their opinion to values closer to those leading the shift in opinion. This process is repeated in order to gain statistical significance and, as a result, it provides the probability distribution of nodes eager to change the opinion and adhere to the new politics, $P(u)$.

Note that the opinion variable for each node, ${u}_{i}$, is real, any number between $0$ and $1$ is accessible. This is consistent with the situation we are trying to describe where most of the people may not have a clear opinion on how to face a pandemic and, thus, their opinion is not 0 neither 1. In fact, a value of 0.5 may indicate that those persons may behave differently depending on the occasion or simply decide to reduce their interactions but not completely.

### Epidemiological model and coupling with opinion probability distribution

**Figure 2:**
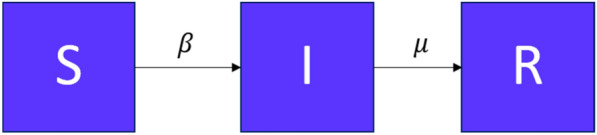
Scheme of the SIR model.

Our epidemiological model is based on the classic SIR model^28^ and considers three different states for the population: susceptible (S), infected (I) and recovered or removed (deceased) individuals (R) with the transitions as sketched in Fig. 2. This model has been extensively analyzed in the previous literature.

Here $\beta$ represents the probability of infection and $\mu$ the probability of recovering. We assume that recovered individuals gain immunity and therefore cannot be infected again or simply die and, obviously, cannot become susceptible again. We consider an extended model to account for the epidemic propagation where each node interacts with others in order to spread the virus. In this context we consider that each node belongs to a complex network whose topology describes the physical interactions between individuals. The meaning of node here is a single person or a set of individuals acting as a close group (i.e. families). The idea is that the infected nodes can spread the disease with a chance $\beta$ to each of its connections with susceptible individuals, thus $\beta$ becomes a control parameter of how many individuals an infected one can propagate the disease to at each time step. Then, each infected individual has a chance $\mu$ of being recovered from the disease.

We add complexity to this model by incorporating two important issues. First, we consider a network of nodes interacting with each other. The way this interaction occurs describes the mobility of our individuals. On the other hand, we include the results of the opinion model via modulations of the infection rate. Those individuals whose opinion is more prone to follow government policies are more likely to be protected and, thus, their infection rate should be lowered (and vice versa). In this way, this simple epidemic model is able to incorporate both the mobility of the population as well as the adhesion to government policies of social distancing. In the following, we explain the details of mathematically incorporating these effects into the epidemic model.

A first order approach to a human mobility network is the Watts-Strogatz model^29^, given its ability to produce a clustered graph where nearest nodes have higher probability of being interconnected while keeping some chances of interacting with distant nodes (as in an Erdös-Renyi random graph^30^). According to this model, we generate a graph of $N$ nodes, where each node is initially connected to its $k$ nearest neighbors in a ring topology and the connections are then randomly rewired with distant nodes with a probability ${p}_{rewire}$. The closer this probability is to 1 the more resembling the graph is to a fully random network while for ${p}_{rewire}=0$ it becomes a purely diffusive network. If we relate this ring-shaped network with a spatial distribution of individuals, when ${p}_{rewire}$ is small the occurrence of random interactions with individuals far from our circle of neighbors is highly severed, mimicking a situation with strict mobility restrictions where we are only allowed to interact with the individuals from our neighborhood. This feature makes the Watts-Strogatz model an even more suitable choice for the purposes of our study since it allows us to impose further mobility restrictions to our individuals in a simple way. On the other hand, the effects of clustering in small-world networks with epidemic models are important and have been already studied^31–34^.

The network is initialized setting an initial number of nodes as infected while the rest are in the susceptible state and, then, the simulation starts. At each time step, the chance that each infected individual spreads the disease to each of its susceptible connections is evaluated by means of a Monte Carlo method^35^. Then, the chance of each infected individual being recovered is evaluated at the end of the time step in the same manner. This process is repeated until the pool of infected individuals has decreased to zero or a stopping criterion is achieved.

**Figure 3:**
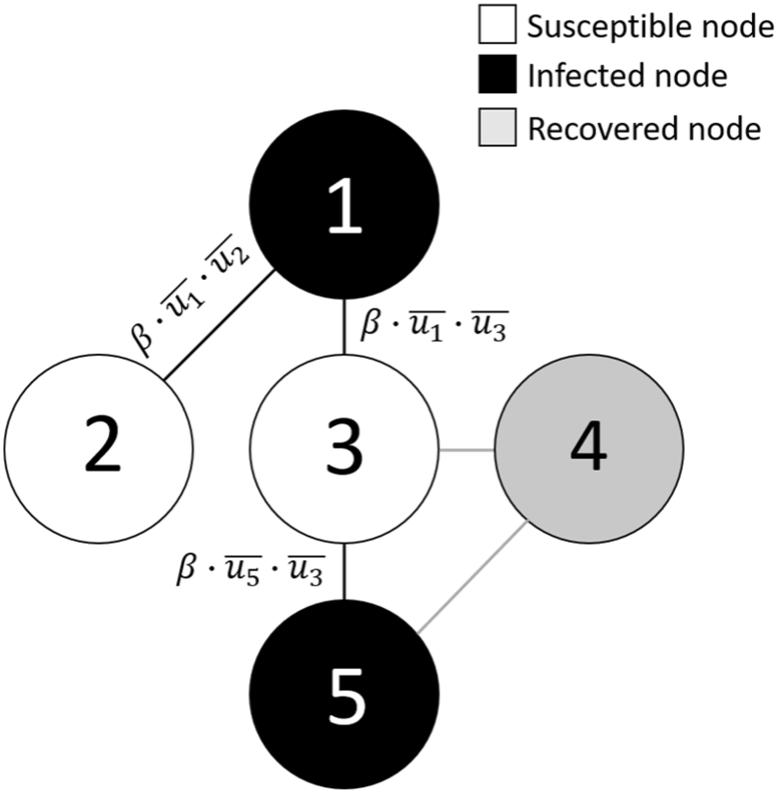
Diagram of the infection process in the network. Black links account for possible infections with weight ${\beta }_{ij}^{^{\prime}}=\beta \cdot \stackrel{-}{{u}_{i}}\cdot \stackrel{-}{{u}_{j}}$.

The following step in our modelling is to include the opinion model results from the previous section in the epidemic spread model just described. As we introduced above, the opinion distribution calculated with the previous opinion model will modulate the infection rate $\beta$ of each node or person and, thus, will strongly influence the evolution of the pandemic First, from the outcome of the opinion model $u$, we build a probability density $P(\stackrel{-}{u})$ where $\stackrel{-}{u}=1-u$ represents the disagreement with the externally given opinion. Here $1$ means that the person is in total disagreement with the government policies and, thus, will not follow the social distancing rules. On the contrary, a value of $0$ means complete adhesion to these rules resulting in an additional protection. These opinion values are randomly assigned to each of the nodes in the Watts-Strogatz network following the disagreement distribution $P(\stackrel{-}{u})$. The idea is to simulate a setup in which each node of the mobility network represents an individual with its own opinion, and interacts with the rest of the nodes accordingly to this opinion. The next step is then to introduce a modified $\beta$ parameter, which varies depending on the said opinion value of the nodes. This is understood in terms of a weighted network modulated by the opinions: it is more likely that an infection occurs between two rogue individuals (higher value of the disagreement variable $\stackrel{-}{u}$) rather than between two individuals who agree with the government confinement policies ($\stackrel{-}{u}$ almost zero or very close to zero). We introduce, then, the weight ${\beta }_{ij}^{^{\prime}}=\beta \cdot \stackrel{-}{{u}_{i}}\cdot \stackrel{-}{{u}_{j}}$, which accounts for the effective probability of infection between an infected node $i$ and a susceptible node $j$. In the worst scenario, $\stackrel{-}{{u}_{i}}=\stackrel{-}{{u}_{j}}=1$ and ${\beta }_{ij}^{^{\prime}}=\beta$, while in the case where one of the nodes has null disagreement with distancing policies, $\stackrel{-}{{u}_{i}}=0$, the infection probability becomes zero. At each time step of the simulation, the infection chances are evaluated accordingly to the value ${\beta }_{ij}^{^{\prime}}$ of the connection and the process is repeated until the pool of infected individuals has decreased to zero or the stopping criterion is achieved. In Fig. 3, we exemplify this process through a network diagram, where white, black and grey nodes represent susceptible, infected and recovered individuals respectively. Black connections account for possible infections with chance ${\beta }_{ij}^{^{\prime}}$.

To account for further complexity, this approach could be extrapolated to more complex epidemic models already presented in the literature^8,12,36^. Nevertheless, for the sake of illustration, this model still preserves the main features of an epidemic spread without adding the additional complexity to account for real situations such as the COVID19 case.

**Figure 4:**
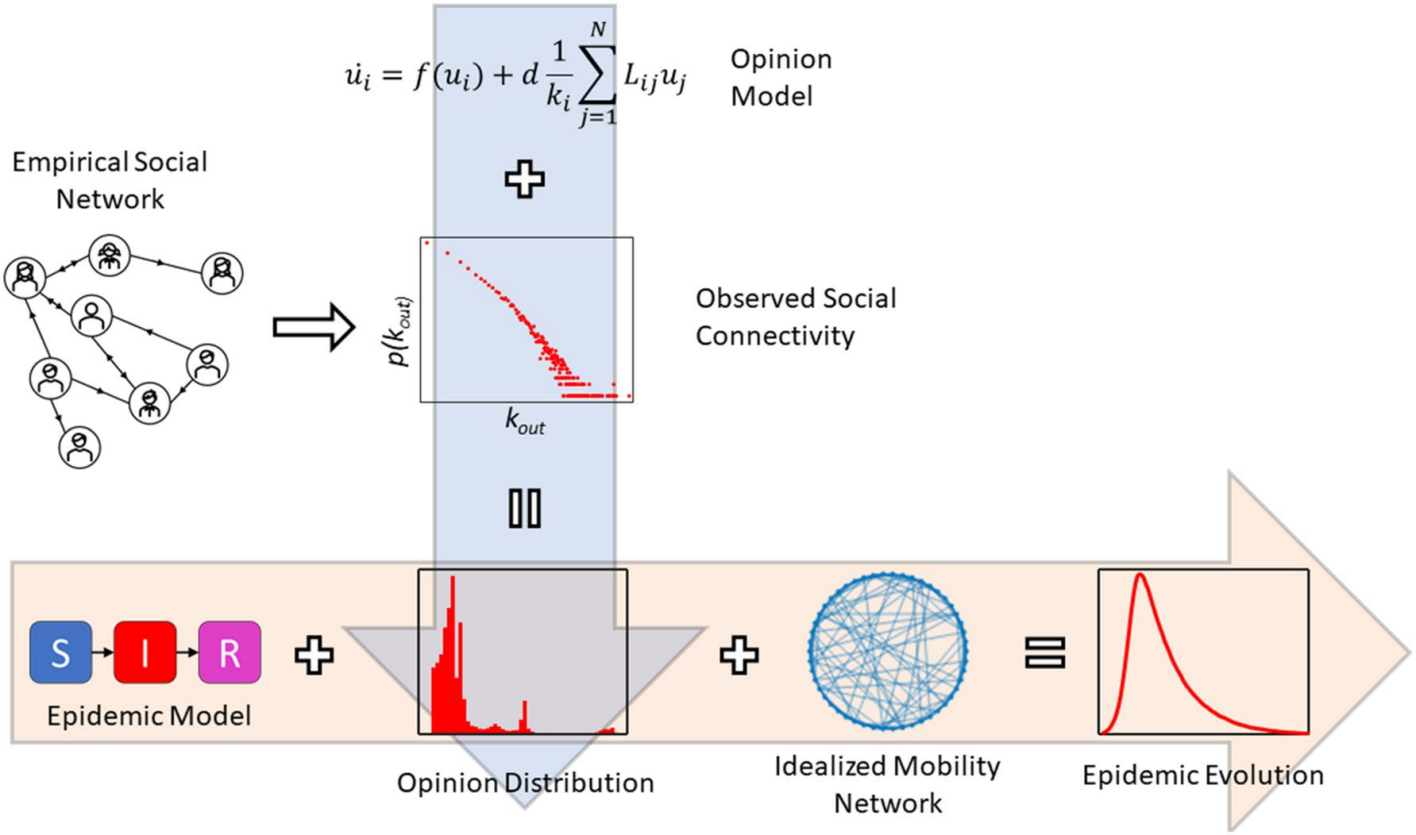
Workflow of the simulations. The empirical social network is obtained from microblogging. This network is introduced in an opinion model (blue arrow) that provides an opinion distribution function statistically describing the eagerness of the population to follow new government policies (social distance policies). The last step (orange arrow) incorporates this opinion model as modulation of the infection rate in an epidemic model.

For clarity, Fig. 4 contains a summary of the methodology explained along this section, from the development of the opinion distribution, to coupling of the models and the final outcome of the epidemiological model.

## Results

In this section, the tools introduced in the Methods section are used to analyze the effect of the individual opinion in the pandemic evolution. The protocol followed is summarized in Fig. 4. Once the social network is measured from experimental evidence, the connectivity of the network is observed. This social connectivity is then introduced into an opinion model (vertical bluish arrow in Fig. 4) that produces a distribution of opinions that in our context will measure the population adherence to the government policies of social distance. This distribution tells the total amount of people that is eager to follow the government social distancing policies and, in general, how each individual positions him or herself with these policies. The protocol used assumes that those rogue individuals are more likely to be infected or infect others and this is directly translated into individual infection rates. The horizontal orange line in Fig. 4 shows the last part of the protocol. The opinion distribution is coupled with a simple epidemic model via the individual infection rates and, considering a mobility network, it is possible to analyze the pandemic evolution.

### Social network

**Figure 5:**
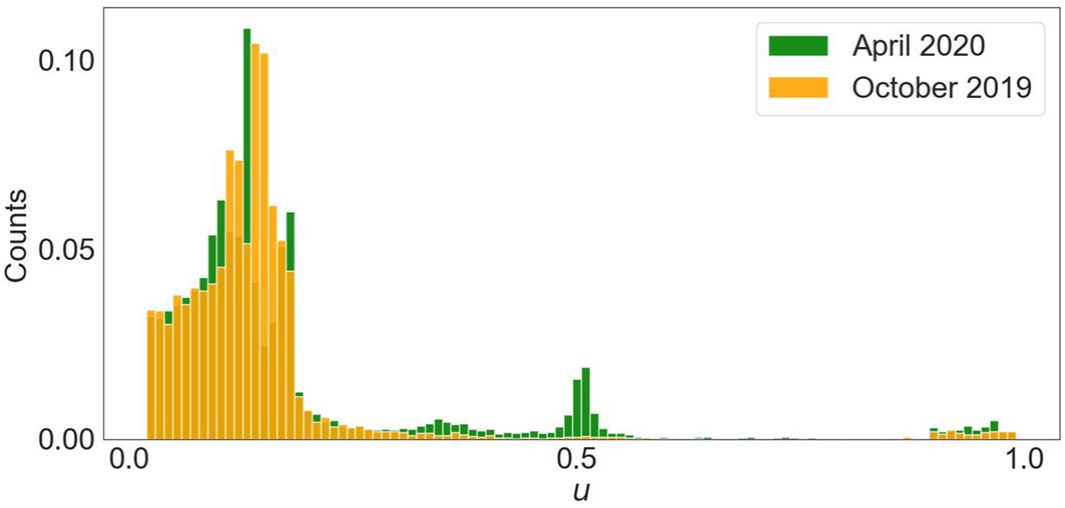
Distribution of the concentrations ${u}_{i}$ (normalized to 1) for the Twitter network from October 2019 (orange) and April 2020 (green) for a r = 30% of the initial accounts in the state 1 with a 10% of noise (*A* = 0.0001, *B* = 0.01, *g* = 0.0001, × 0 = 0.01, *d* = 20,000).

Following the previous protocol, we run the opinion model considering the two social networks analyzed. Figure 5 shows the distribution of the final states of the $u$ variable for the October’19 network (orange) and the April’20 network (green) when the new opinion is introduced in a 30% of the total population (r = 30%). Note that the opinion variable $u$ describes how likely is a person to follow government social distancing policies. Different percentages of the initial population *r* were considered but the results are equivalent (see figure S1 in the supplementary information).

Direct inspection of Fig. 5 clearly shows that the population in April’20 is more eager to follow the new opinion (political guidelines) comparing with the situation in October’19. In the pandemic scenario (network of April’20) it is noticeable that larger values of the opinion variable, ${u}_{i}$, are achieved corresponding to the period of the quarantine. Preferential states are also observed around ${u}_{i}=0,$ ${u}_{i}=0.5$ and ${u}_{i}=1$. Note that the network of April’20 allows to change opinions more easily than in the case of October’19. Also note that the October’19 network corresponds with a polarized situation in the Spanish society as discussed above and the degree of consensus in anything was very limited (and this is reflected in the opinion distribution obtained).

### Opinion biased epidemic model

**Figure 6:**
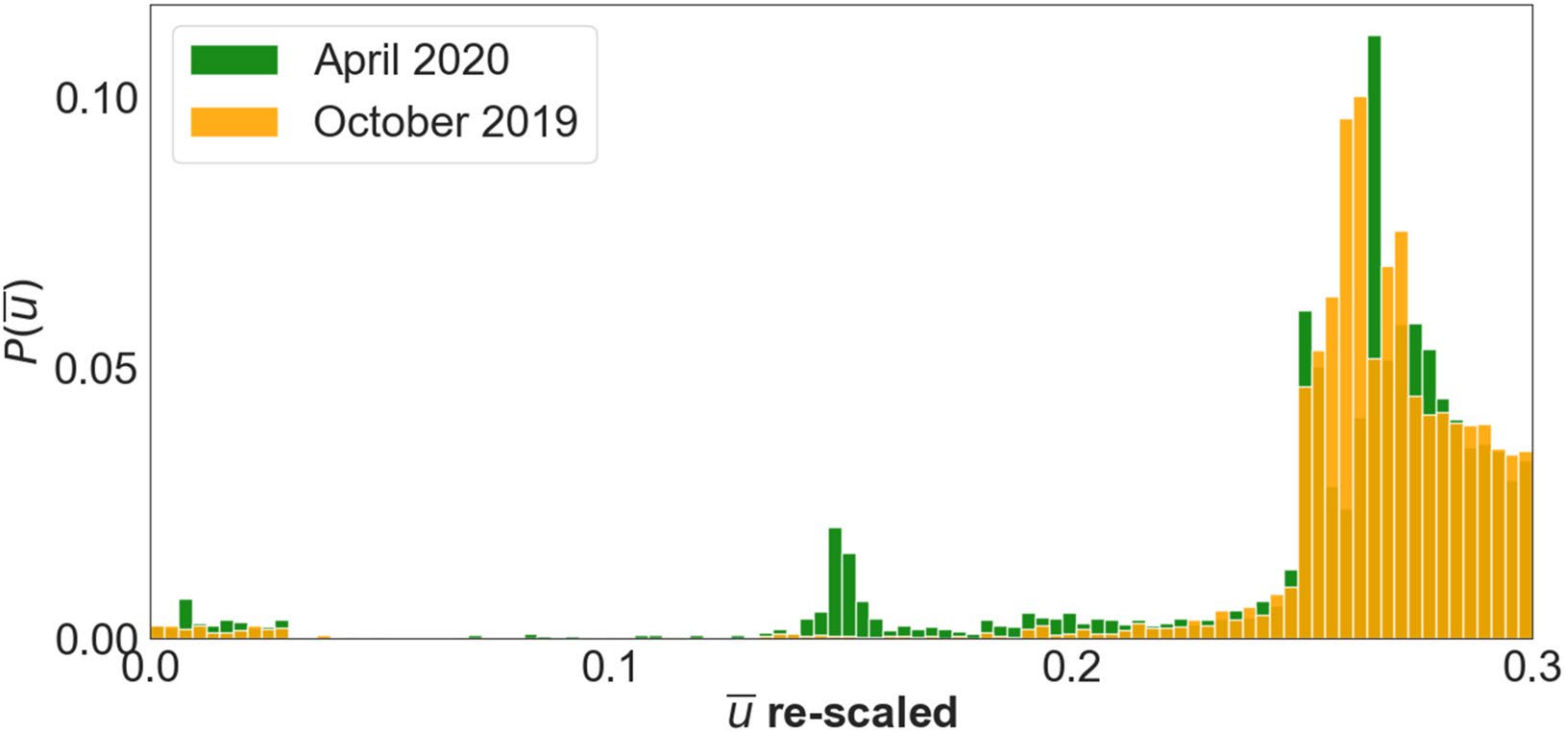
Probability densities of the variable $\stackrel{-}{u}=1-u$ constructed from the distributions of Fig. 5 and rescaled to the values between 0 and 0.3 to account for a heavily restricted mobility.

During the sanitary crisis in Spain, the government imposed heavy restrictions on the mobility of the population. To better account for this situation, we rescaled the probability density of disagreement opinions $P(\stackrel{-}{u})$ (where $\stackrel{-}{u}=1-u$ measured the disagreement with government social distance policies) to values between 0 and 0.3, leading to the probability densities of Fig. 6. From here on, we shall refer to this maximum value of the rescaled probability density as the ***cutoff*** imposed to the probability density. Note that this probability distribution is directly included into the mobility model as a probability to interact with other individuals, thus, this cutoff means that the government policy is enforced reducing up to a 70% of the interactions and the reminder 30% is controlled by the population decision to adhere to the official opinion.

**Figure 7:**
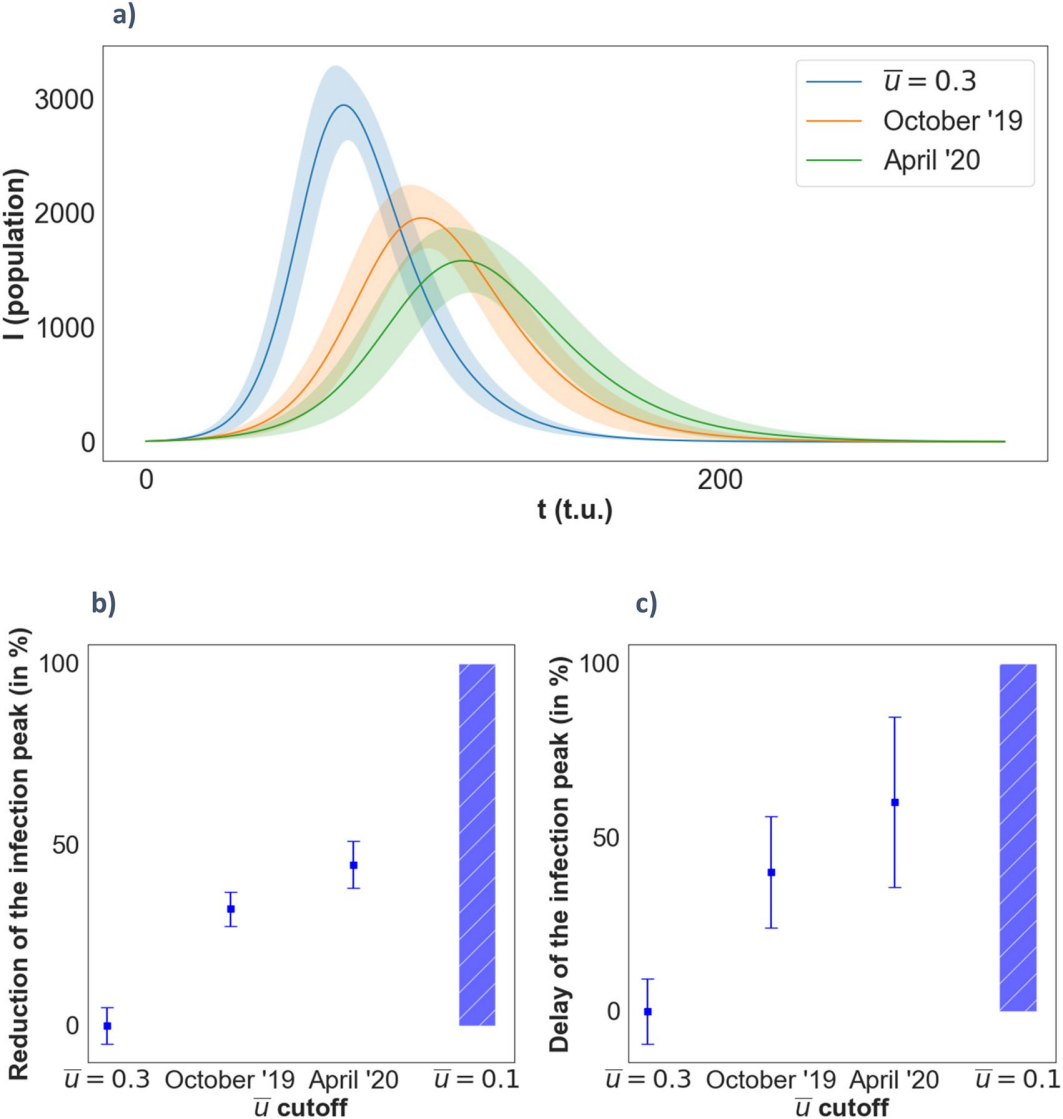
**(a)** Evolution of the number of infected individuals with time for the three opinion models considered. **(b)** Reduction in % of the infected individuals on the peak of the curve with respect to the model with fixed opinion $\stackrel{-}{u}=0.3$. **(c)** Time delay in % of the infection peak. Error bars represent the standard deviation of a sample of peak statistics obtained across several simulations with the same parameters, but different random configurations of the network’s adjacency matrix. ($N=\mathrm{10,000}, \beta =0.05, \mu =0.06, {p}_{rewire}=0.25)$.

In Fig. 7 we summarized the main results obtained from the incorporation of the opinion model into the epidemiological one. We established four different scenarios: for the first one we considered a theoretical situation where we imposed that around the 70% of the population will adopt social distancing measures, but leave the other 30% in a situation where they either have an opinion against the policies or they have to move around interacting with the rest of the network for any reason (this means, $\stackrel{-}{u}=0.3$ for all the nodes). In contrast to this situation we introduce the opinion distribution of the social networks of April’20 (population fully aware of the dramatic pandemic situation) and October’19 (a convulsive political moment marked by polarization). Finally, we consider another theoretical population where at least 90% of the population will adopt social distancing measures (note that in a real situation, around 10% of the population occupies essential jobs and, thus, are still exposed to the virus). However, for the latter the outbreak of the epidemic does not occur so there is no peak of infection. Note that the first and the last ones are completely in-silico scenarios introduced for the sake of comparison. For all the scenarios, and unless something else is specified, the initial number of infected individuals is set to 5 and are distributed randomly along the network with no specific criteria. The size of the mobility networks is set to $N=10000$.

Figure 7a shows the temporal evolution of the infected population in the first three of the above scenarios. The line in blue shows the results without including an opinion model and considering that a 70% of the population blindly follows the government social distance restrictions (probably by fear of fines or other type of coercivity measurements) while the reminding 30% continue interacting as usual. Orange line shows the evolution including the opinion model with the probability distribution derived as in October’19. The green line is the evolution of the infected population considering the opinion model derived from the situation in April’20. Note that the opinion model stated that the population in April’20 was more eager to follow changes in the opinion than in October’19, and this is directly reflected in the curves in Fig. 7a. Also note that as the population becomes more conscious and decides to adhere to the restriction-of-mobility policies, the maximum of the infection curve differs in time and its intensity is diminished. This figure clearly shows that the state of the opinion inferred from the social network analysis strongly influences the evolution of the epidemic. In fact, from these results we can infer that a society that follows the social distancing policies is more likely to delay the pandemic wave and diminish its intensity thus reducing the pressure on the health system.

The results from the first theoretical case (blue curve) clearly show that the disease reaches practically all the rogue individuals (around the 30% of the total population that we set with the rescaling of the probability density), while the other two cases with real data show that further agreement with the given opinion results in flatter curves of infection spread. We have analyzed both the total number of infected individuals on the peaks and its location in time of the simulation, but, since our aim is to highlight the incorporation of the opinion model we show in Fig. 7b,c the values of the maximum peak infection as well as the delay introduced in achieving this maximum scaled with the corresponding values of the first case (blue line) for all the cases analyzed. We see that the difference on the degree of adhesion of the social networks outcomes a further 12% reduction approx. on the number of infected individuals at the peak, and a further delay of around the 20% in the time at which this peak takes place. Note that for the April’20 social network, a reduction of almost the 50% of individuals is obtained for the peak of infection, and a similar value is achieved for the time delay of the peak. This clearly reflects the fact that a higher degree of adhesion to the government social-distance policies is important to flatten the infection curve (i.e. that the probability distribution of disagreement, $P(\stackrel{-}{u})$, is more weighted in the lower values close to $\stackrel{-}{u}=0$) . Finally, in the latter theoretical scenario, where we impose a cutoff of $\stackrel{-}{u}=0.1$, the outbreak of the epidemic does not occur, and thus there is no peak of infection. This is represented in Fig. 7b,c as a dash-filled bar indicating the absence of the said peak.

**Figure 8:**
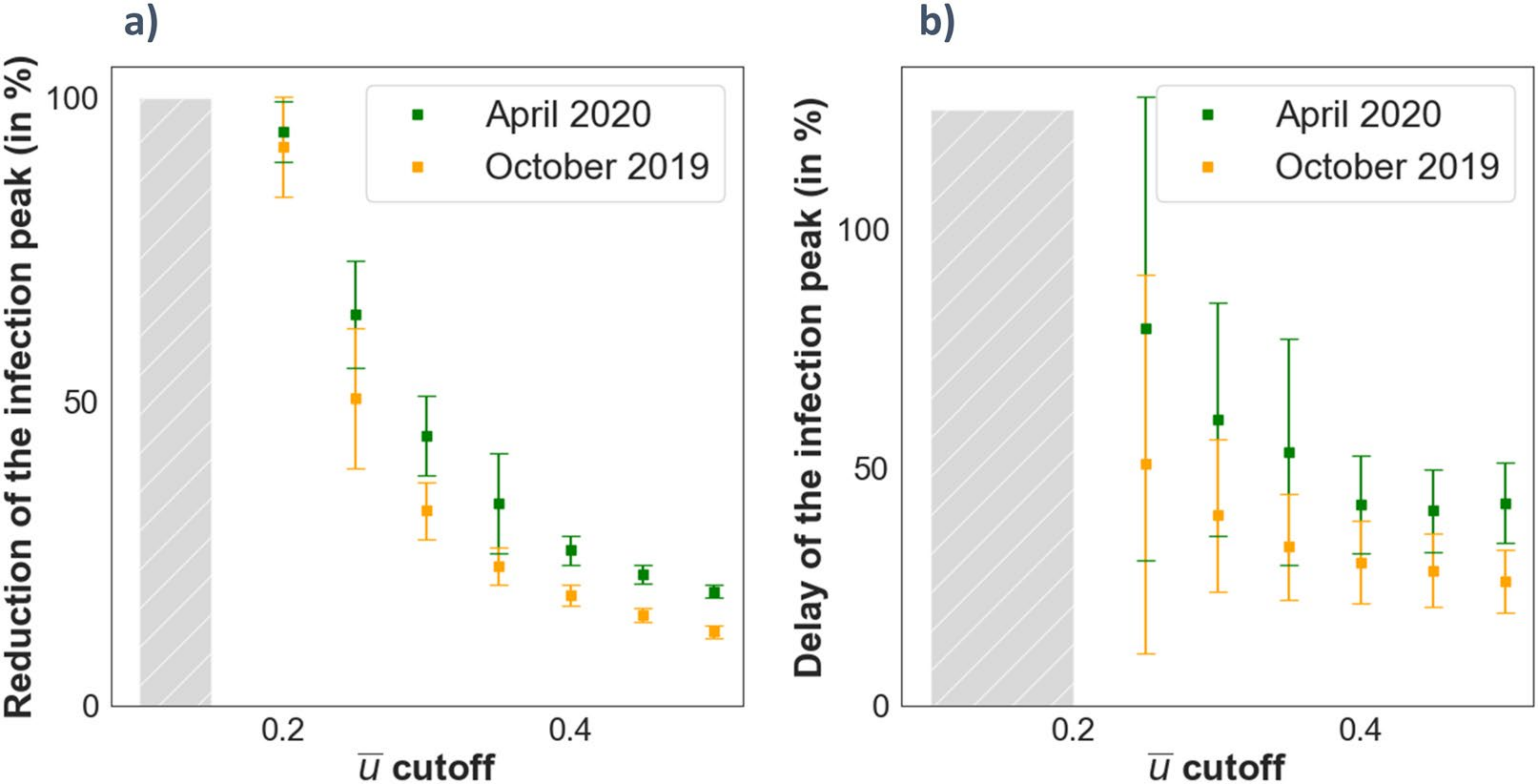
Infection peak statistics for different values of the cutoff of $\stackrel{-}{u}$ and for both the opinion models (October’19 in yellow and April’20 in green). **(a)** Reduction of the maximum in the infection curve scaled with the corresponding maximum of the least favorable case (theoretical scenario where all nodes have the opinion of the cutoff). **(b)** Delay of the maximum in the infection curve scaled with the corresponding time for the maximum of the least favorable case. Again, the dash-filled bar represents the absence of an infection peak, and the error bars represent the standard deviation of a sample of peak statistics obtained across several simulations. See figure S3 on the supplementary information for the time evolution of the infected individuals of some of the points shown here. *(*$N=10000, \beta =0.05, \mu =0.06, {p}_{rewire}=0.25)$*.*

Changing the condition on the cutoff imposed for the disagreement variable $\stackrel{-}{u}$ can be of interest to model milder or stronger confinement scenarios such as the different policies ruled in different countries. In Fig. 8 we show the infection peak statistics (maximum of the infection curve and time at maximum) for different values of the cutoffs and for both social opinion networks. In both cases, the values are scaled with those from the theoretical scenario with all individuals having their opinion at the cutoff value. Both measurements (Fig. 8a,b) are inversely proportional to the value of the cutoff. This effect can be understood in terms of the obtained probability densities. For both networks (October’19 and April’20) we obtained that most of the nodes barely changed their opinion, and thus for increasing levels on the cutoff of $\stackrel{-}{u}$ these counts dominate on the infection processes so the difference between both networks is reduced. On the other hand, this highlights the importance of rogue individuals in situations with increasing levels of confinement policies since for highly contagious diseases each infected individual propagates the disease rapidly. Each infected individual matter and the less connections he or she has the harder is for the virus to spread along the exposed individuals. Note that for all the scenarios, the social network of April’20 represents the optimum situation in terms of infection peak reduction and its time delay. It is particularly interesting the case for the cutoff in $\stackrel{-}{u}=0.2$. All simulations run for this cutoff show an almost non-existent peak. This is represented on Fig. 8a with almost a reduction of the 100% of the infection peak (the maximum value found on the infection curve was small but not zero) and the value of the time delay (Fig. 8b) is included in the shaded region since this infection curve was almost flat. Something similar occurs for the cutoff in $\stackrel{-}{u}=0.25$, which explains the large error seen in Fig. 8b. Note that this value of the cutoff, $\stackrel{-}{u}=0.2$, constitutes by itself an actual threshold bellow which no infection peak is observed.

**Figure 9:**
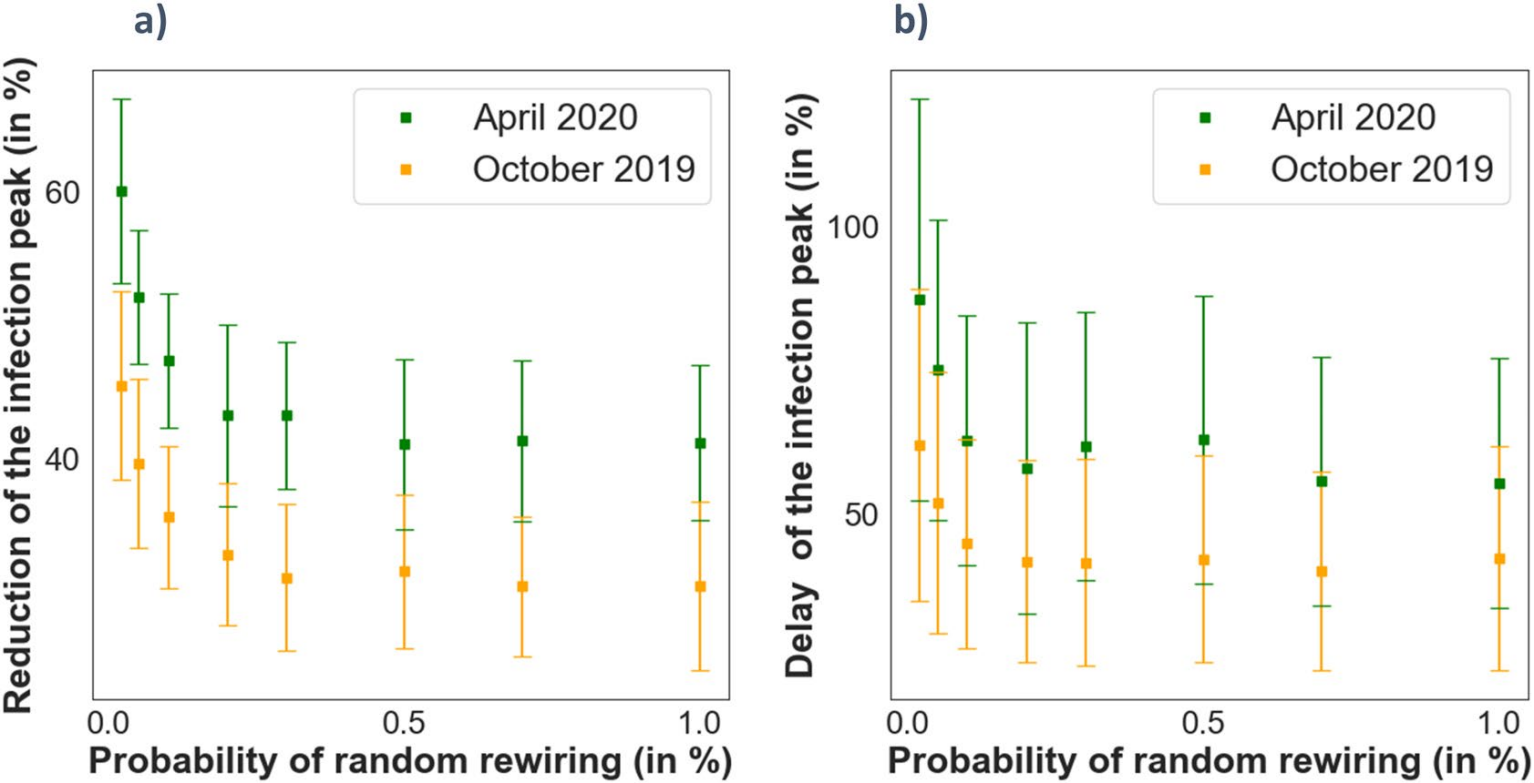
Peak statistics of the infection curves for different values of the rewiring probability of the Watts-Strogatz mobility model, and for both opinion scenarios October’19 and April’20. See figure S4 on the supplementary information for the time evolution of the infected individuals of some of the points shown here. ($N=\mathrm{10,000}, \beta =0.05, \mu =0.06, \stackrel{-}{u }\mathrm{cutoff}=0.3)$

As discussed in the previous section, we are considering a Watts-Strogatz model for the mobility network. This type of network is characterized by a probability of rewiring (as introduced in the previous section) that stablishes the number of distant connections for each individual in the network. All previous results were obtained considering a probability of rewiring of 0.25. Figure 9 shows the variation of the maximum for the infection curve and time for the maximum versus this parameter. The observed trend indicates that the higher the clustering (thus, the lower the probability of rewiring) the more difficult is for the disease to spread along the network. This result is supported by previous studies in the field, which show that clustering decreases the size of the epidemics and in cases of extremely high clustering, it can die out within the clusters of population^31,34^. This can be understood in terms of the average shortest path of the network^21^, which is a measure of the network topology that tells the average minimum number of steps required to travel between any two nodes of the network. Starting from the ring topology, where only the nearest neighbors are connected, the average shortest path between any two opposite nodes is dramatically reduced with the random rewiring. Remember that these new links can be understood as short-cuts or long-distance connections within the network. Since the infection process can only occur between active links between the nodes, it makes sense that the propagation is limited if less of these long-distance connections exist in the network. The average shortest path length decays extremely fast with increasing values of the random rewiring, and thus we see that the peak statistics are barely affected for random rewirings larger than the 25%. If one is interested on further control of the disease, the connections with distant parts of the network must be minimized to values smaller than this fraction. Regarding the performance of both opinion biased epidemic cases, we found again a clear difference between the two of them. In the April’19 case, the outcome of the model present always a more favorable situation to control the expansion of the epidemic independently on the rewiring probability, stating the importance of the personal adherence to isolation policies in controlling the evolution of the epidemic.

## Discussion

We have parametrized the social situation of the Spanish society at two different times with the data collected from a social media based on microblogging (*Twitter*). The topology of these networks combined with a simple opinion model provides us with an estimate of how likely this society is to follow new opinions and change their behavioral habits. The first analysis presented here shows that the social situation in October 2019 differs significantly from that of April 2020. In fact, we have found that the latter is more likely to accept opinions or directions and, thus, follow government policies such as social distancing or confining. Of course, April 2020 corresponds with the worst moment of the first epidemic wave and the population was eager to follow the directions given by authorities. On the other hand, October 2019 marks a moment of total polarization in the Spanish society marked for a second call to general elections in a short time and after the politicians were unable to reach a consensus to produce a stable government. Our results show that an epidemic wave reaching Spain with an opinion distribution such as that in October 2019 may result in worst conditions for the health system and, probably, a larger death toll.

The output of these opinion models was used to tune the mobility in an epidemic model aiming to highlight the effect that the social ‘mood’ has on the pandemic evolution. The histogram of opinions was directly translated into a probability density of people choosing to follow or not the directions, modifying their exposedness to being infected by the virus. Although we exemplify the results with an over-simplified epidemic model (SIR), the same protocol can be implemented in more complicated (and probably more realistic) epidemic models. We show that the partial consensus of the social network, although non perfect, induces a significant positive impact on the infection curve, and that this impact is quantitatively stronger in the network of April 2020. Our results are susceptible to be included in more sophisticated models used to study the evolution of the COVID19.

Although plenty of effort has been destinated to give more realism to the models and include social response into epidemiological models through, e.g., mobility networks based on real demographic data or exhaustive control strategies within the models, taking into account the actual mood or social state of the population measured from social media has not been explored much in the literature. We propose here a way to monitor, almost in real time, the mood of the society and, therefore, include it in a dynamic epidemic model that is biased by the population eagerness to follow the government policies.

Further analysis of the topology of the social network may also provide insights of how likely the network can be influenced and identify the critical nodes responsible for the collective behavior of the network.

## Supplementary Information

Supplementary Information.
