# Supplementary material for: Incorporating social opinion in the evolution of an epidemic spread
Source: Sci Rep. 2021 Jan 19;11:1772. doi: 10.1038/s41598-021-81149-z (PMC7815732; doi:10.1038/s41598-021-81149-z)
Supplement: Supplementary file 1 — Supplementary Information. [file 41598_2021_81149_MOESM1_ESM.pdf]

Supplementary information

Incorporating social opinion in the evolution of an epidemic spread

Alejandro Carballosa, Mariamo Mussa Juane and Alberto P. Muñuzuri

Institute CRETUS. Group of Nonlinear Physics. Fac. Physics. University of Santiago de Compostela. 15782 Santiago de Compostela, Spain

Supplementary contents

1. List of Hashtags used to build up the social networks considered.

2. Opinion distributions depending on the initial number of nodes with different opinion.

3. Opinion biased epidemic model

1. List of Hashtags used to build up the social networks considered.

The list of hashtags used to construct both networks is in Table 1 for the October’19 case (column on the left) and for the April’20 scenario (right column). All hashtags used were neutral in the sense of political bias or age meaning.

October’19	April’20
#eleccionesgenerales28a	#CuidaAQuienTeCuida
#eldebatedecisivolasexta	#EsteVirusLoParamosUnidos
#PactosARV	#QuedateConESP
#RolandGarros	#SemanaEnCasaYoigo
#NiUnaMenos	#QuedateEnCasa
#selectividad2019	#Superviviente2020
#AnuncioEleccions28Abril	#AutonomosAbandonados
#BlindarElPlaneta	#Renta2019
#DiaMundialDeLaBicicleta'	#EnCasaConSalvame
#EmergenciaClimatica27S'	#diamundialdelasalud
	#CuarentenaExtendida
	#AsiNonUvigo
	#AhoraTocaLucharJuntos
	#House_Party
	#EnCasaConSalvame
	Apoyare_a_Sanchez
	Pleno_del_Congreso
	Viernes_de_Dolores

Table 1: List of hashtags used to construct the networks.

In order to check the statistical accuracy and relevance of our networks, we considered different scenarios with more or less subnets (each subnet corresponding with a single hashtag) and estimate the exponent of the scale-free-network fit. This result is illustrated in Figure S1a for the October'19 case and in Figure S1b for the April'20 case. Note that as the number of subnets (hashtags) is increased, the exponent converges.

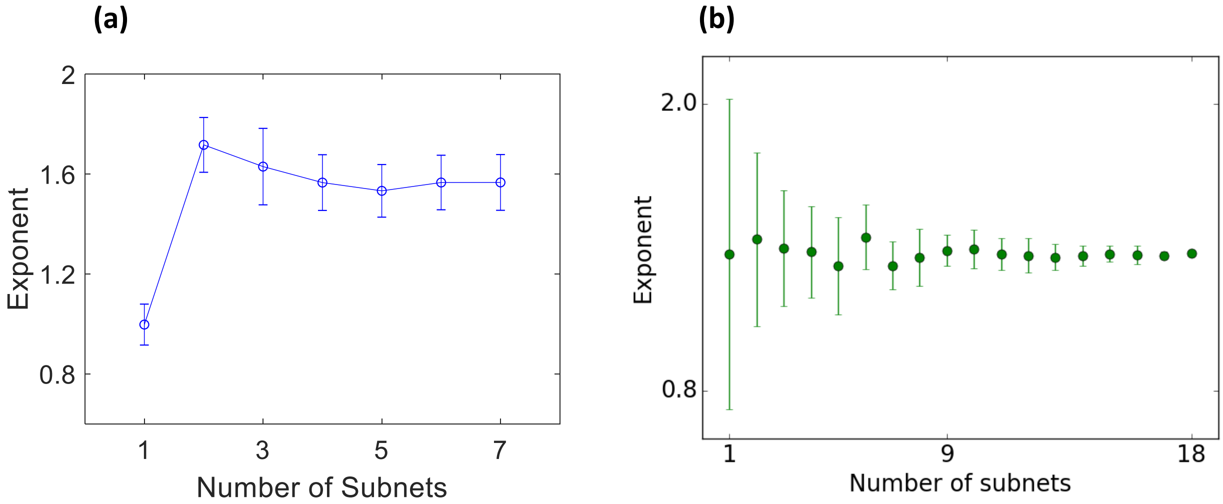

Figure S1. Variation of the exponent versus the number of subnets considered (a) October 2019. (b) April 2020 exponent of the scale free distribution. Each one of the exponents was calculated merging 10 combinations of $2, 3, \ldots, N-1$ subnets. The error bars are the standard deviation. For 1 subnet all the exponents were calculated and for N subnets just one combination is possible so that non deviation is shown.

2. Opinion distributions depending on the initial number of nodes with different opinion.

Distribution of the final states of the u variable for the October'19 network (orange) and the April'20 network (green) when the new opinion is introduced by three different percentages of the total population (r parameter) is shown in figure S2. Note that in all cases the results are qualitatively equivalent and, once included in the opinion model, the results are similar.

a) $r = 0.2$

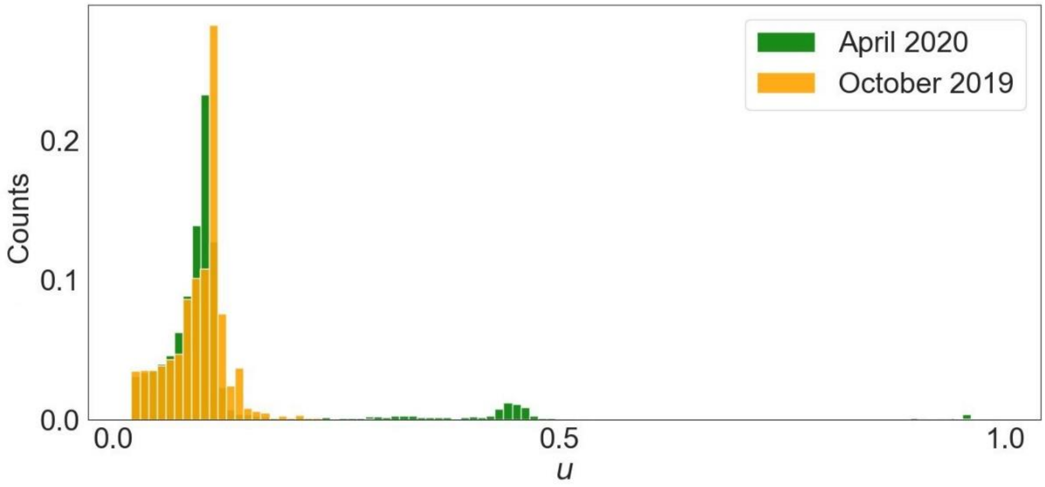

b) $r = 0.3$

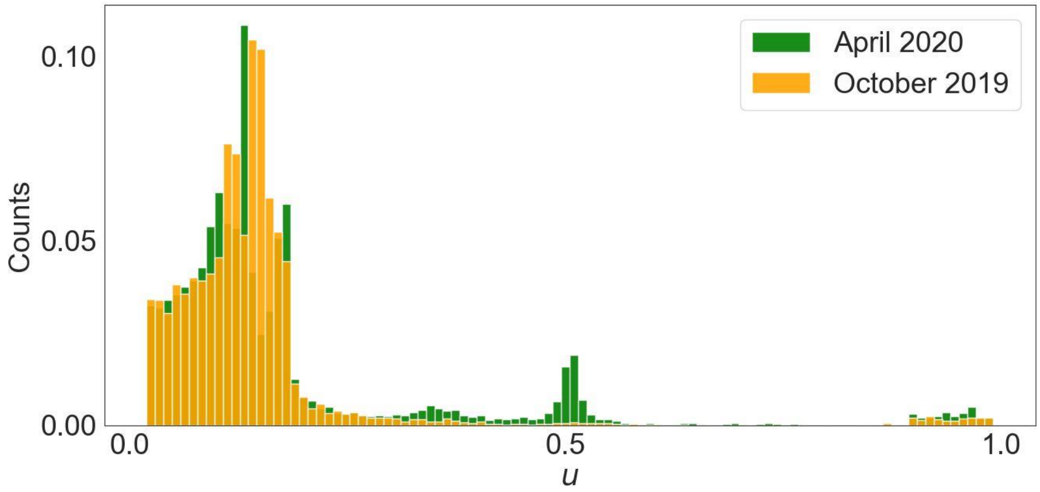

c) $r = 0.4$

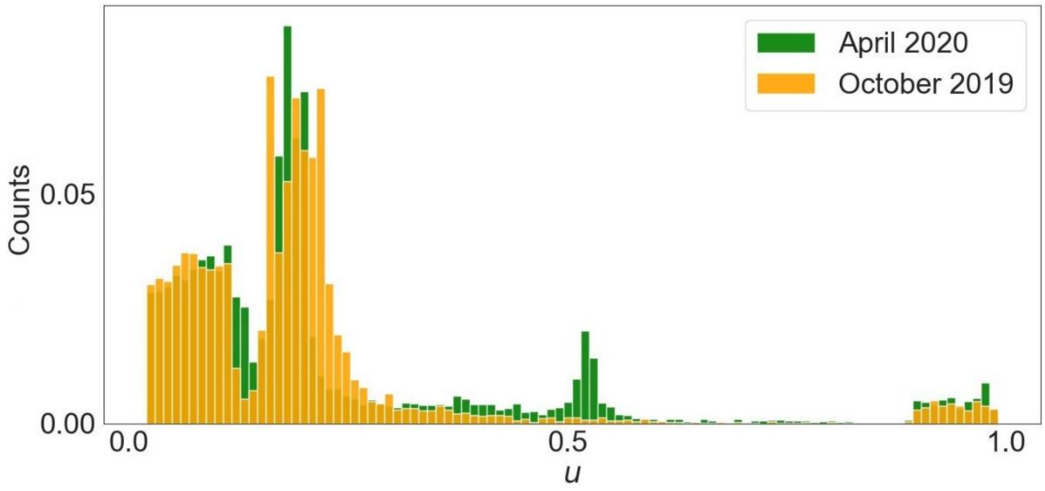

Figure S2. Distribution of the concentrations u_i (normalized to 1) for the Twitter network from October 2019 (orange) and April 2020 (green) for r=20% (a), r=30% (b) and r=40% (c) of the initial accounts in the state 1 with a 10% of noise (A=0.0001, B=0.01, g=0.0001, x0=0.01, d=20000).

3. Opinion biased epidemic model

Figure S3 shows the evolution of the number of infected individuals with time for the epidemic model biased with the opinion model of April 2020. Unless specified differently, the initial number of infected individuals was set to 5 and the size of the mobility nodes in the network to $N = 10000$. Results for different values of the $\bar{u}$ cutoff are shown. Note how for a disagreement variable value $\bar{u} = 0.2$ the peak of infection vanishes, and the epidemic dies out due to its lack of ability to spread among the nodes. On the other hand, Figure S4 shows for different values of the cutoff on $\bar{u}$, the comparison between the three cases presented in the main text (see figure 7): the theoretical scenario where the opinion is fixed on the cutoff value for all the nodes, and the epidemic model biased with the opinions of October '19 and April '20 scenarios. See how the difference between the theoretical scenario and the opinion biased models diminishes with growing values of the cutoff value on $\bar{u}$

Finally, Figure S5 shows the effect that higher values of the rewiring probability of the Watt-Strogatz model has in the time evolution of the infected individuals. As shown in the main text, lower values of the rewiring probability has an important impact on the peak of infection, while values above $p_{rewiring} = 0.3$ barely change the statistics on the said peak, or fall within the error of the measurements.

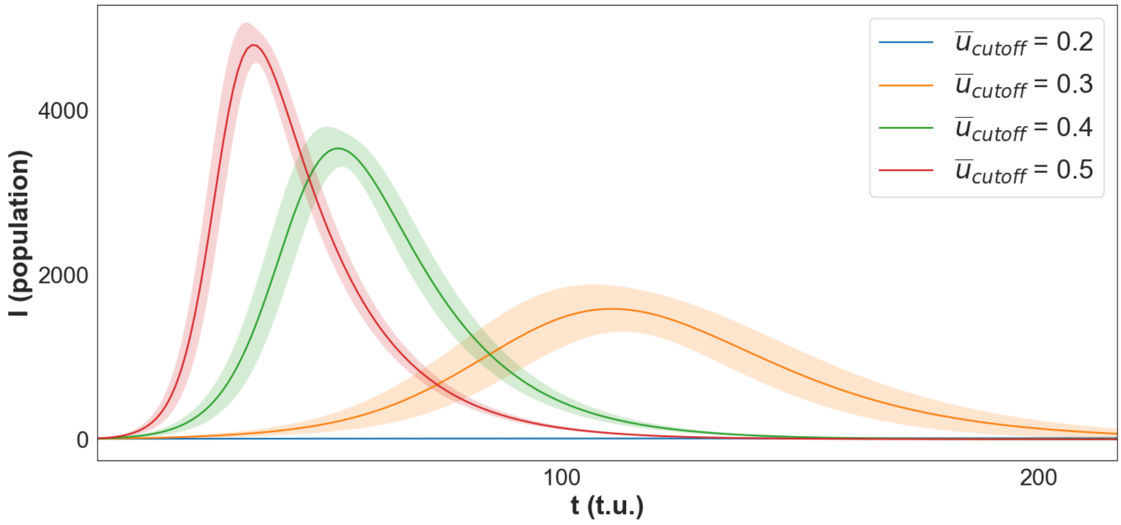

Figure S3. Evolution of the number of infected individuals with time for the epidemic model biased with the April'20 social network and for different values of the cutoff on $\bar{u}$. ($N = 10000$, $\beta = 0.05,\ \mu = 0.06,\ p_{rewire} = 0.25$)

a) $\bar{u} = 0.25$

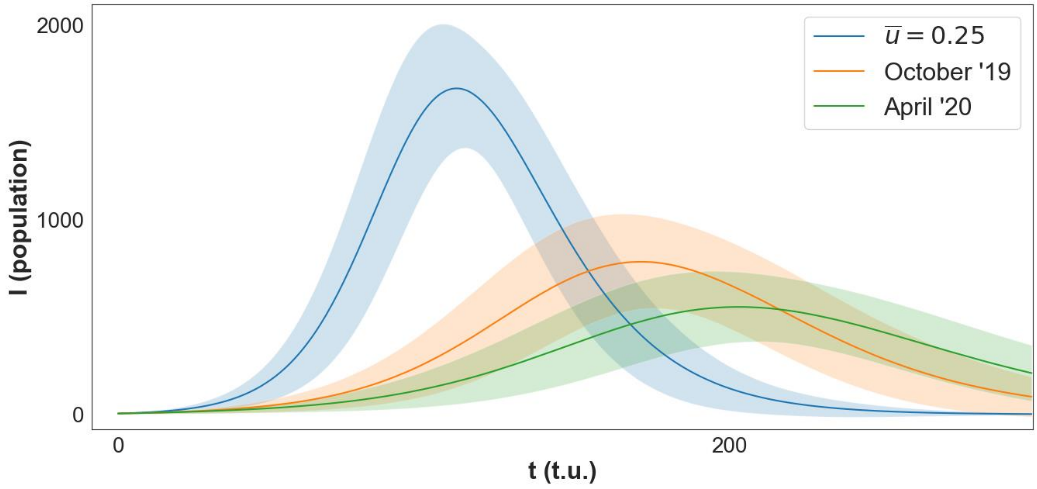

b) $\bar{u} = 0.4$

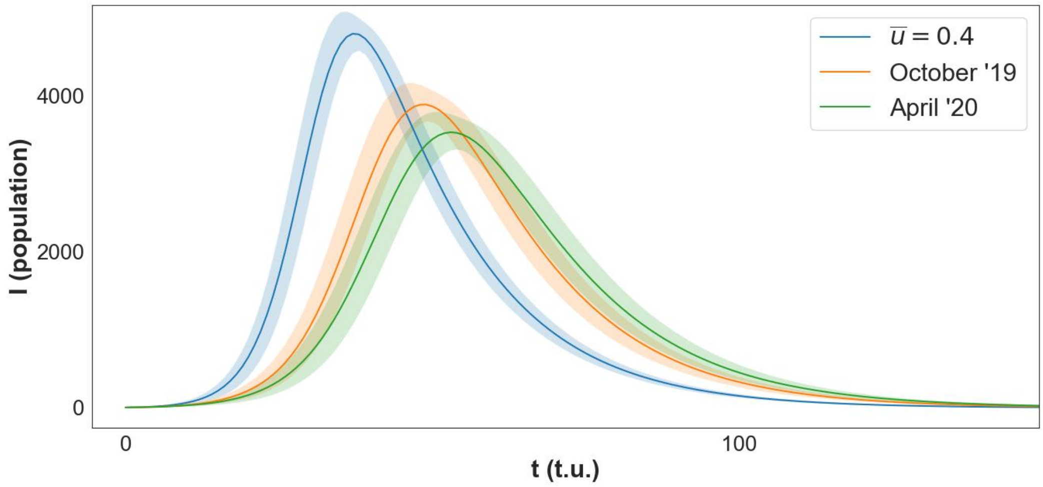

c) $\bar{u} = 0.5$

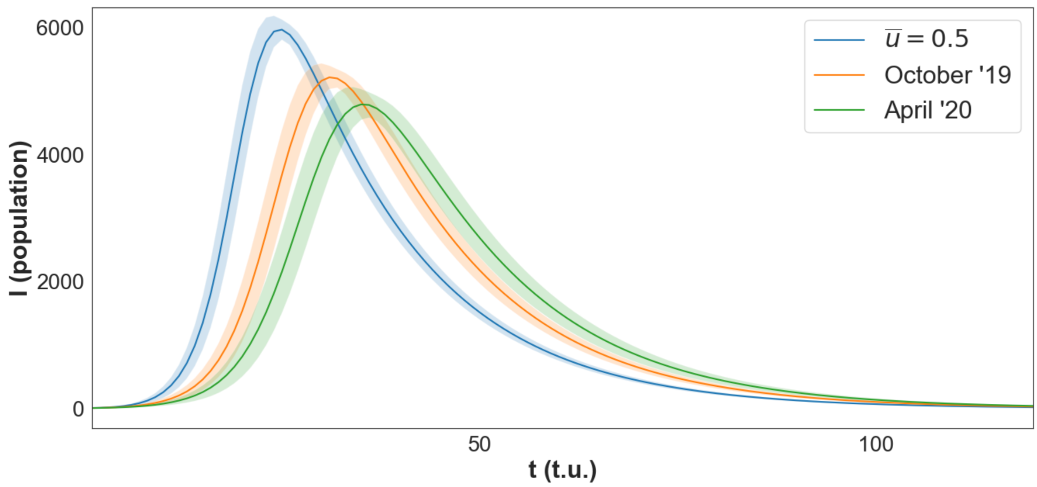

Figure S4. Evolution of the number of infected individuals with time for the three opinion models considered for three different values of the cutoff on $\bar{u}$: a) $\bar{u}$=0.25, b) $\bar{u}$=0.4 and c) $\bar{u}$=0.5. ($N = 10000,\ \beta = 0.05,\ \mu = 0.06,\ p_{rewire} = 0.25$)

a)

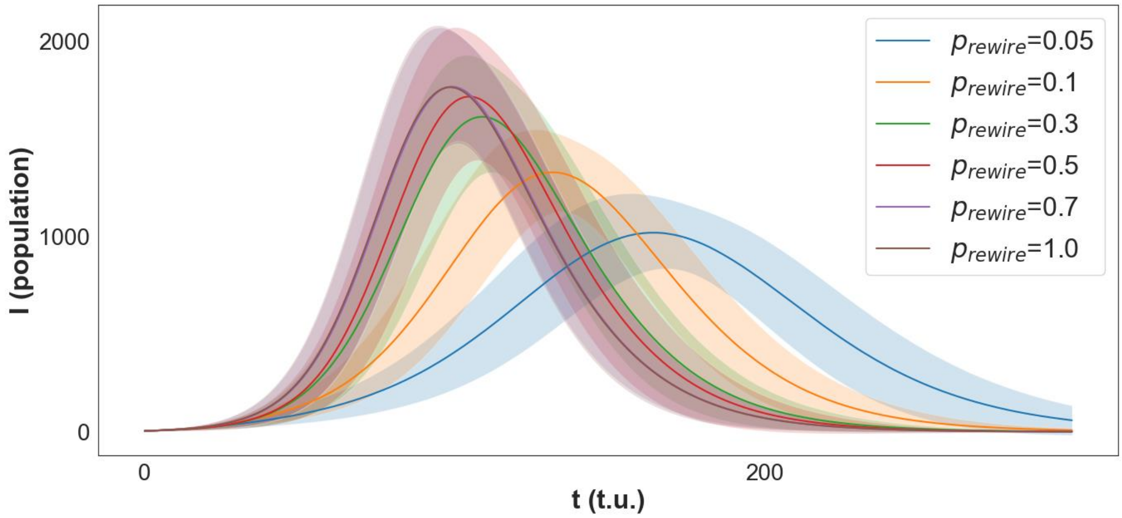

b)

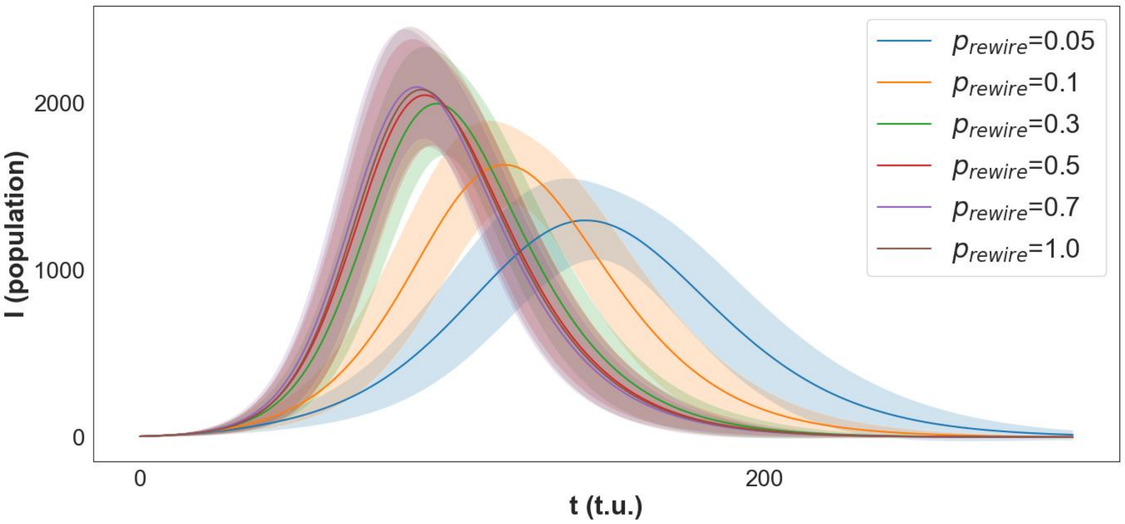

Figure S5. Evolution of the number of infected individuals with time for the epidemic models biased with the April'20 social network (a) and the October'19 social network (b), for different values of the rewiring probability of the Watt-Strogatz network model. ($N = 10000$, $\beta = 0.05$, $\mu = 0.06$, $\bar{u}\ cutoff = 0.3$)
